# Psychological capital and classroom management self-efficacy during teaching practicum: a mixed-methods study of pre-service English teachers

**DOI:** 10.3389/fpsyg.2026.1822494

**Published:** 2026-05-28

**Authors:** Manolya Sağlam

**Affiliations:** Department of English Language Teaching, Faculty of Education, Biruni University, Istanbul, Türkiye

**Keywords:** classroom management self-efficacy, mixed-methods, pre-service teachers, psychological capital, resilience, teaching practicum

## Abstract

Teaching practicum represents a critical phase in teacher education, where pre-service teachers encounter real classroom challenges that require both pedagogical competence and psychological readiness. Grounded in positive psychology, this study examines the relationship between psychological capital and classroom management self-efficacy among pre-service English teachers. An explanatory sequential mixed-methods design was employed. Quantitative data were collected from 55 pre-service teachers using validated scales, followed by qualitative data obtained through open-ended reflective responses to explain the quantitative findings. Pearson correlation analyses revealed a strong, positive, and statistically significant relationship between psychological capital and classroom management self-efficacy (*r* = 0.71, *p* < 0.001). Among Psychological capital components, resilience showed the strongest association (*r* = 0.74), despite having comparatively lower mean scores. Qualitative findings provided explanatory insight into how psychological resources are experienced in emotionally demanding practicum contexts. Participants emphasized the importance of emotional regulation, persistence, and coping with classroom disruptions, highlighting the role of resilience in managing real classroom challenges. The findings are interpreted as a “resilience paradox,” suggesting that resilience may become particularly salient under practicum-related stress, while operating alongside other psychological resources. Overall, the study supports a context-sensitive understanding of psychological capital as a dynamic system contributing to classroom management self-efficacy. These results underscore the importance of integrating psychological capacity-building into teacher education to better support pre-service teachers in navigating the demands of practicum.

## 1 Introduction

Teaching practicum constitutes a critical phase in initial teacher education, during which pre-service teachers are required to translate theoretical knowledge into practice while simultaneously managing classroom dynamics, emotional demands, and professional role expectations (Ji et al., 2022). In this period, the classroom becomes not only an instructional space but also a psychologically intensive environment characterized by uncertainty, stress, and emotional labor. As highlighted in previous research, pre-service teachers often experience practicum as a form of “professional reality shock,” which may challenge their sense of competence and professional identity (Pendergast et al., 2011).

For pre-service teachers who are in the practicum phase, the classroom is not only a setting in which instructional skills are demonstrated but also a developmental context characterized by highlevels of stress, uncertainty, and emotional demands (Bandura, 1997; Ji et al., 2022). Within this context, teachers’ beliefs about their capability to manage classrooms play a contextually salient role in shaping instructional effectiveness and emotional wellbeing.

Teacher self-efficacy has long been recognized as a key psychological construct predicting classroom processes, instructional quality, and teacher resilience (Tschannen-Moran et al., 1998; Zee and Koomen, 2016). However, recent scholarship suggests that self-efficacy alone may be insufficient to explain how teachers cope with the complex emotional and behavioral challenges of real classrooms, particularly during the early stages of professional development such as practicum (Mu et al., 2024). Previous research has emphasized that psychological resources such as teacher self-efficacy and resilience enhance classroom effectiveness, strengthen teachers’ stress-coping capacities, and promote more consistent classroom management practices (Bandura, 1997; Tschannen-Moran et al., 1998). Recent research has consistently demonstrated that psychological resources, particularly teacher self-efficacy and resilience, are significantly associated with classroom effectiveness, enhanced stress-coping capacities, and more adaptive instructional practices (Heng and Chu, 2023; Mu et al., 2024; Mieres-Chacaltana et al., 2025).

Building on positive psychology, psychological capital has emerged as an integrative framework for understanding how developable psychological resources support individuals’ functioning under pressure. Psychological capital is commonly defined as a higher-order construct comprising self-efficacy, hope, optimism, and resilience, representing individuals’ positive psychological capacities that are measurable and open to development (Luthans et al., 2006). Recent studies indicate that psychological capital is meaningfully associated with teachers’ professional wellbeing, emotional experiences, and efficacy beliefs (Zhou and Zheng, 2022; Youssef-Morgan and Luthans, 2015).

Given the emotionally demanding nature of the teaching profession, the psychological capital levels of teachers—particularly pre-service teachers who are at the early stages of their careers—play a decisive role in shaping classroom behaviors and classroom management performance (Luthans and Youssef, 2004; Tösten, 2015).

From a psychological perspective, classroom management should not be conceptualized merely as a pedagogical skill but rather as a psychologically grounded competence requiring emotional regulation, stress tolerance, and adaptive coping. Empirical evidence suggests that classroom management self-efficacy is one of the most anxiety-sensitive domains of teacher self-beliefs during practicum and is closely linked to teachers’ emotional responses to classroom disruptions (Slater and Main, 2020). Accordingly, pre-service teachers’ perceived capability to manage classroom behavior is shaped not only by instructional knowledge but also by the psychological resources they can mobilize under stress.

Among the components of psychological capital, resilience appears to play a particularly critical role in classroom management contexts. Resilience enables individuals to recover from setbacks, maintain emotional balance, and persist in goal-directed behavior despite adverse conditions (Masten and Reed, 2002). Recent meta-analytic evidence confirms that teacher resilience and teacher self-efficacy are moderately to strongly related and tend to develop together across teaching contexts (Mu et al., 2024). Moreover, longitudinal and cross-sectional studies demonstrate that resilience-based psychological resources predict teacher self-efficacy both directly and indirectly through emotion regulation mechanisms (Zhang and Zhang, 2025; Mieres-Chacaltana et al., 2025). Within this framework, psychological capital provides a critical psychological foundation that enables pre-service teachers to cope with classroom discipline problems, communication challenges, and role ambiguity (Akçay, 2011; Luthans et al., 2006).

Despite the growing body of research on psychological capital and teacher self-efficacy, important gaps remain. First, much of the existing literature on psychological capital and teacher self-efficacy has primarily focused on in-service teachers and professional or organizational outcomes (Avey et al., 2009; Zhou and Zheng, 2022), while comparatively fewer studies have examined pre-service teachers in practicum contexts. Recent research also indicates that studies focusing specifically on pre-service teachers’ self-efficacy and related psychological constructs remain relatively limited and context-dependent (Baimukhambetova et al., 2026; Karas et al., 2026). Second, although classroom management self-efficacy is recognized as a critical determinant of early teaching success, its relationship with psychological capital has received relatively limited attention in studies employing mixed-methods designs that integrate statistical associations with teachers’ lived experiences in practicum classrooms.

Addressing these gaps, the present study investigates the relationship between psychological capital and classroom management self-efficacy among pre-service English teachers during teaching practicum using an explanatory sequential mixed-methods design. By combining quantitative analyses with qualitative insights, the study aims to clarify not only whether psychological capital predicts classroom management self-efficacy, but also how pre-service teachers experience and enact psychological resources—particularly resilience when facing classroom management challenges in authentic practicum settings. Drawing on Bandura’s (1997) self-efficacy theory, individuals’ beliefs in their capabilities are shaped not only by knowledge and skills but also by their psychological resources and their ability to cope with challenging situations. In the context of teaching practicum, where emotional demands and uncertainty are high, psychological capital may function as a critical set of personal resources that supports pre-service teachers’ ability to regulate emotions, cope with stress, and sustain effective classroom management. Accordingly, psychological capital is expected to play a key role in shaping classroom management self-efficacy in real teaching contexts.

In light of the theoretical considerations discussed above, particularly the role of psychological capital in supporting adaptive responses to emotionally demanding teaching contexts, the present study aims to systematically investigate the extent, structure, and experiential manifestations of the relationship between psychological capital and classroom management self-efficacy. Accordingly, the following research questions guide the present investigation:

RQ1: To what extent is psychological capital associated with classroom management self-efficacy among pre-service English teachers during teaching practicum?

RQ2: Which components of psychological capital most strongly predict classroom management self-efficacy?

RQ3: How do pre-service English teachers explain the role of psychological capital in managing classroom challenges during practicum?

## 2 Conceptual framework

### 2.1 Psychological capital as a developable resource in teaching practicum

Recent research in educational psychology emphasizes that effective teaching is shaped not only by pedagogical knowledge and instructional skills but also by teachers’ psychological resources, particularly in demanding professional contexts such as the teaching practicum (Ji et al., 2022; Zhou and Zheng, 2022). Within this perspective, psychological capital is conceptualized as a measurable and developable psychological capacity that supports individuals’ performance, motivation, and adaptive functioning under challenging conditions (Luthans et al., 2006).

Psychological capital differs from traditional forms of capital by focusing on individuals’ future-oriented psychological potential rather than solely on existing competencies or skills (Luthans and Youssef, 2004). It is defined as a higher-order construct comprising self-efficacy, hope, optimism, and resilience, which together form a synergistic system of positive psychological resources (Luthans et al., 2006). Rooted in the positive psychology movement, Psychological capital emphasizes the strengthening of individuals’ adaptive capacities rather than the remediation of deficits, thereby aligning with contemporary approaches to teacher development (Seligman and Csikszentmihalyi, 2000; Gable and Haidt, 2005).

In the context of the teaching practicum, pre-service teachers encounter authentic classroom demands characterized by emotional strain, uncertainty, and classroom management challenges. These environments function as critical developmental settings in which psychological resources become particularly salient, as novice teachers must cope with discipline issues, communication difficulties, and role ambiguity while simultaneously constructing their professional identities. Within this context, psychological capital provides a valuable framework for understanding how pre-service teachers regulate their emotions, sustain motivation, and adapt to complex classroom realities beyond pedagogical knowledge alone (Erden, 2025).

### 2.2 The role of resilience within psychological capital in practicum contexts

Although psychological capital comprises four interrelated components, resilience is not conceptualized as a dominant or exclusive factor in the present framework. Rather, it is treated as a contextually salient psychological resource that becomes more visible and functionally significant under conditions of stress and uncertainty, such as those experienced during the teaching practicum.

Resilience refers to individuals’ psychological capacity to recover, adapt, and maintain equilibrium in the face of adversity, stress, and uncertainty (Luthans, 2002; Masten and Reed, 2002). In educational settings, individuals with higher levels of resilience tend to demonstrate more flexible, calm, and solution-oriented responses when confronted with discipline problems and communication difficulties (Norman, 2006; Luthans et al., 2008). Within practicum contexts, where pre-service teachers frequently encounter emotional pressure, classroom management challenges, and instructional unpredictability, resilience appears to play a particularly important role in sustaining adaptive functioning.

This interpretation is consistent with contemporary evidence suggesting that resilience is especially relevant in emotionally demanding teaching contexts for supporting teachers’ efficacy beliefs and professional functioning (Mu et al., 2024; Mieres-Chacaltana et al., 2025). From a psychological process perspective, resilience can be understood as a resource that supports the functioning of other Psychological capital components under stress. While self-efficacy contributes to confidence in managing classroom situations, hope facilitates goal-directed persistence, and optimism shapes adaptive interpretations of classroom events, resilience may influence the extent to which these resources can be effectively mobilized when classroom conditions threaten emotional stability and instructional continuity (Zhang and Zhang, 2025).

**Figure1:**
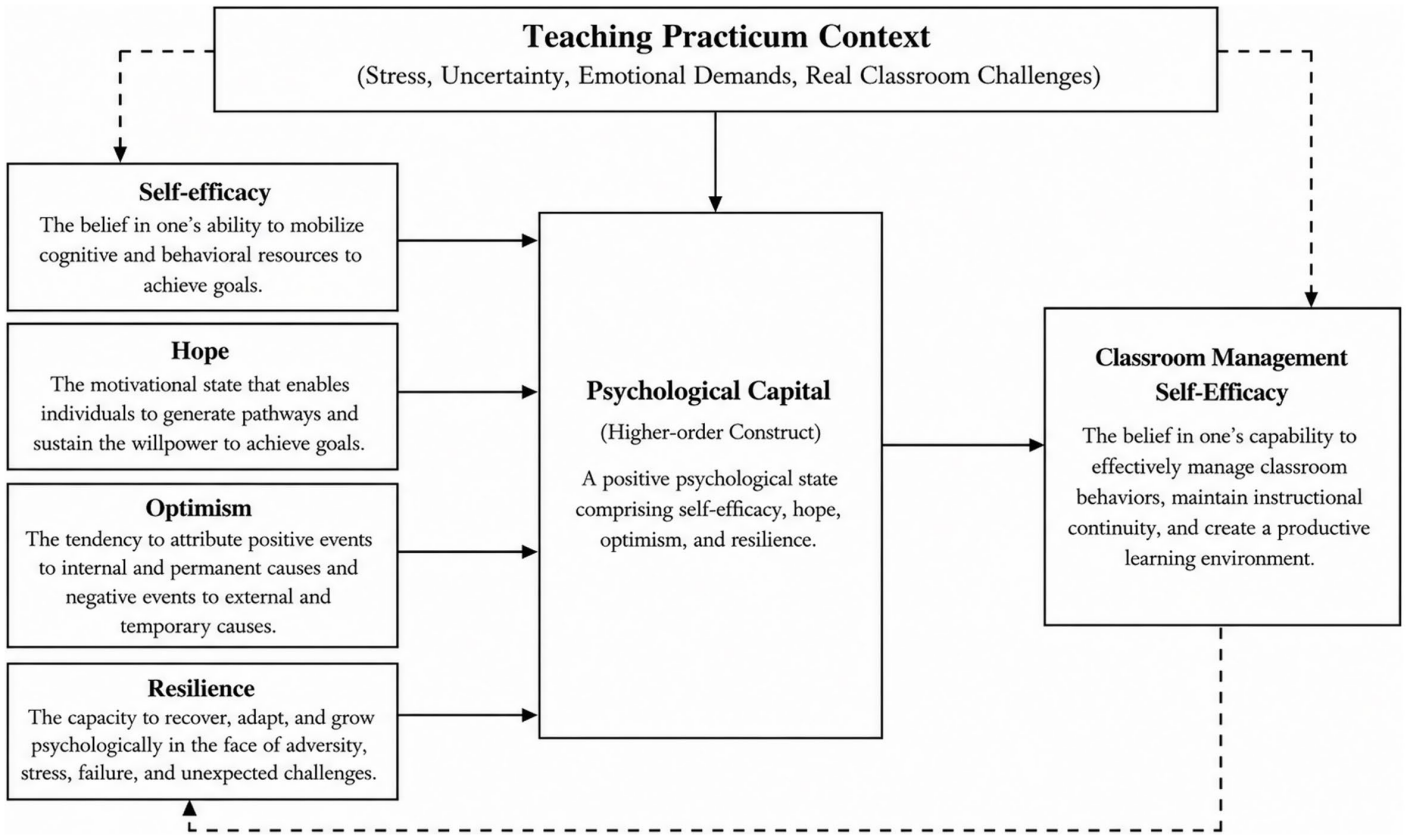
Conceptual model of psychological capital and classroom management self-efficacy in teaching practicum contexts.

Accordingly, within the present framework, resilience is neither treated as a peripheral subdimension nor positioned as a singular determining mechanism. Instead, it is conceptualized as a contextually prominent resource that supports the dynamic interplay of psychological capital components in demanding practicum environments. In this sense, psychological capital operates as an integrated system of interrelated resources, within which resilience may become more salient under conditions of stress, contributing to the translation of psychological capacities into classroom management self-efficacy. This conceptualization is visually represented in Figure 1.

This figure illustrates the conceptual framework underlying the present study. Psychological capital is represented as a higher-order construct comprising self-efficacy, hope, optimism, and resilience. Within the teaching practicum context—characterized by stress, uncertainty, and emotional demands—these components collectively contribute to pre-service teachers’ classroom management self-efficacy. The model emphasizes the integrated and context-dependent nature of psychological resources, while also reflecting the finding that resilience may become a particularly salient resource under conditions of stress, supporting the effective mobilization of other psychological capacities.

### 2.3 Classroom management self-efficacy as a psychologically grounded outcome

Classroom management self-efficacy refers to teachers’ beliefs in their capability to organize classroom environments, manage student behavior, and sustain instructional order in the face of disruptions (Slater and Main, 2020). Although classroom management is often discussed within pedagogical discourse, it is fundamentally psychological in nature, as it reflects teachers’ confidence in regulating both classroom processes and their own emotional responses. Consistent with the findings and theoretical positioning of the original study, classroom management self-efficacy among pre-service teachers is particularly sensitive during practicum, as real classroom experiences may challenge confidence developed through coursework or simulated teaching environments. Emotional overload, anxiety, and perceived loss of control can undermine self-efficacy even when pedagogical knowledge is adequate, underscoring the importance of psychological resources that support emotional regulation and stress tolerance. Within the proposed framework, classroom management self-efficacy is conceptualized as the outcome of psychological resource mobilization under classroom stress. Psychological capital constitutes the broader resource base, while its components—including self-efficacy, hope, optimism, and resilience—interact dynamically to support adaptive functioning. In emotionally demanding practicum contexts, resilience may become particularly salient due to its association with stress tolerance and recovery; however, it operates in conjunction with other psychological resources rather than as a singular determining mechanism.

### 2.4 Integrated conceptual model

Drawing on the theoretical framework and empirical findings, the present model proposes that psychological capital functions as an integrated system of psychological resources that collectively influence classroom management self-efficacy. Rather than assuming a single dominant pathway, the model suggests that the influence of Psychological capital components may vary depending on contextual demands. In practicum settings characterized by emotional and instructional challenges, resilience may become more salient due to its association with stress tolerance and recovery; however, it operates alongside self-efficacy, hope, and optimism as part of a broader adaptive system. Accordingly, classroom management self-efficacy is conceptualized as a psychologically grounded outcome emerging from the dynamic and context-dependent interplay of multiple psychological resources rather than a single dominant mechanism.

## 3 Methods

### 3.1 Research design

The present study employed an explanatory sequential mixed-methods design, in which quantitative data were collected and analyzed first, followed by qualitative data to explain and contextualize the quantitative findings (Creswell, 2014). This design was selected to examine both the predictive relationships between psychological capital and classroom management self-efficacy and how pre-service teachers experience and enact psychological resources—particularly resilience—during the teaching practicum. This approach aligns with the conceptual framework, which views psychological capital as a system of psychological resources and classroom management self-efficacy as a psychologically grounded outcome. Quantitative analyses examined the relationships among variables, while qualitative findings provided explanatory insight into the underlying psychological processes.

Integration was achieved at the interpretation stage, where qualitative data were used to explain and elaborate quantitative results. A joint display was also developed to enhance the coherence and interpretive validity of the findings. Joint displays are widely recommended in mixed-methods research to enhance transparency and facilitate the integration of qualitative and quantitative evidence (Fetters et al., 2013).

This approach follows established mixed-methods design (Creswell and Clark, 2017) integration strategies that emphasize the importance of connecting statistical patterns with participants’ lived experiences where qualitative inquiry is informed by and extends quantitative results.

### 3.2 Research context

The study was conducted within a four-year English Language Teaching (ELT) undergraduate program at a public university in Türkiye. Teaching practicum constitutes a compulsory component of the final year of the program and involves pre-service teachers engaging in regular classroom teaching at assigned public secondary schools under the supervision of both university mentors and cooperating school teachers.

During the practicum period, pre-service teachers were responsible for planning lessons, managing classroom behavior, implementing instructional activities, and reflecting on their teaching experiences. These responsibilities often exposed participants to classroom management challenges, emotional pressure, and instructional uncertainty, making the practicum context particularly suitable for examining psychological capital, resilience, and classroom management self-efficacy, particularly in line with research highlighting practicum as a high-stress, high-learning context in teacher development (Ji et al., 2022).

### 3.3 Participants

#### 3.3.1 Quantitative phase

The quantitative sample consisted of 55 fourth-year pre-service English teachers (female = 40, male = 15) enrolled in the teaching practicum course during the data collection period. Participants’ ages ranged from 21 to 24 years (*M* = 22.3). Convenience sampling was employed, as participation was based on accessibility and voluntary consent during scheduled practicum sessions. Convenience sampling is commonly used in educational research where access to naturally occurring groups is required (Etikan et al., 2016), and was considered appropriate given the study’s focus on practicum-based experiences within an intact cohort. Inclusion criteria for the sample were: (a) enrollment in the fourth year of the ELT program, (b) active participation in the teaching practicum course, (c) prior completion of core methodology and classroom management courses, and (d) voluntary consent to participate in the study. Exclusion criteria included: (a) not being enrolled in the practicum course at the time of data collection, (b) incomplete or missing questionnaire responses, and (c) withdrawal from participation at any stage of the study. All participants had completed core methodology and classroom management courses prior to practicum, ensuring a comparable level of pedagogical preparation and exposure to practicum-related experiences across the sample.

#### 3.3.2 Qualitative phase

For the qualitative phase, 25 volunteer participants were purposively selected from the quantitative sample. Selection criteria included participants’ willingness to provide reflective responses and the completeness of their quantitative data. The qualitative subsample was used to explain and contextualize quantitative findings, particularly those related to the contextually salient role of resilience in classroom management self-efficacy. Purposeful sampling allowed for the selection of information-rich cases capable of providing deeper insight into the phenomenon under investigation (Patton, 2014, 2015).

### 3.4 Instruments

#### 3.4.1 Psychological capital scale

Psychological capital was measured using the Psychological Capital Scale originally developed by Luthans et al. (2006) and adapted into Turkish by Çetin (2012). The scale consists of 17 items measuring four core components of psychological capital: self-efficacy, hope, optimism, and resilience. Responses were recorded on a 5-point Likert scale ranging from 1 (strongly disagree) to 5 (strongly agree). The scale was administered in Turkish, the participants’ native language. For reporting purposes, an English version of the scale was prepared through expert translation and back-translation procedures conducted by three specialists in English Language Teaching and educational psychology to ensure conceptual and linguistic equivalence. Previous validation studies reported Cronbach’s alpha coefficients above 0.80 for all subdimensions, indicating strong internal consistency (Çetin, 2012). In the present study, the scale demonstrated high internal reliability, with an overall Cronbach’s alpha coefficient of 0.91. Reliability coefficients for the four subdimensions ranged from 0.82 to 0.88, indicating satisfactory internal consistency within the current sample. These reliability values indicate that the scale demonstrates acceptable internal consistency for research purposes (Nunnally and Bernstein, 1994).

#### 3.4.2 Teachers’ classroom management self-efficacy scale

Classroom management self-efficacy was measured using the Teachers’ Classroom Management Self-Efficacy Scale (TCMSES) developed by Slater and Main (2020). The scale consists of 14 items assessing teachers’ perceived capability to establish classroom rules, manage disruptive behaviors, maintain instructional flow, implement non-aversive management strategies, and respond effectively to student misbehavior. Participants responded to the items using a 5-point Likert scale ranging from 1 (strongly disagree) to 5 (strongly agree). The scale was administered in its original English form, as all participants were senior pre-service English language teachers with advanced proficiency in English. The original scale development study reported high internal consistency (*α* = 0.90) and provided evidence for construct validity through Rasch model analyses (Slater and Main, 2020). In the present study, the TCMSES also demonstrated high internal reliability, with a Cronbach’s alpha coefficient of 0.89, indicating strong consistency among items within the current sample. The scale has been widely used in teacher education research and provides robust measurement of classroom management self-efficacy.

#### 3.4.3 Qualitative data collection tool

Qualitative data were analyzed using thematic analysis following the six-phase approach outlined by Braun and Clarke (2006). The analysis was conducted through a systematic and iterative process involving familiarization with the data, initial code generation, theme development, review, definition, and refinement. Both deductive coding, informed by the study’s conceptual framework (e.g., resilience, emotional regulation, stress tolerance), and inductive coding, allowing themes to emerge from participants’ reflections, were employed.

To enhance methodological rigor, the analytic process was conducted in a transparent and iterative manner, ensuring consistency between codes and themes. Special attention was given to maintaining a clear audit trail and ensuring that themes were grounded in participants’ accounts. This approach aligns with contemporary standards for conducting trustworthy thematic analysis, particularly in ensuring coherence, transparency, and analytic rigor (Nowell et al., 2017). Furthermore, the integration of qualitative findings with quantitative results at the interpretation stage was guided by mixed-methods principles, enhancing the overall validity and depth of interpretation (Creswell and Poth, 2016). Analytic decisions were revisited throughout the process to ensure reflexivity and minimize researcher bias.

### 3.5 Data collection procedure

Data collection took place during the middle phase of the teaching practicum semester. After obtaining institutional permission, participants were informed about the purpose of the study and provided written informed consent.

Quantitative instruments were administered during scheduled practicum seminars and required approximately 20–25 min to complete. Following the quantitative phase, participants were invited to provide written reflective responses. The qualitative data collection occurred approximately 2 weeks after the quantitative data collection to allow preliminary quantitative findings to inform the focus of qualitative exploration, consistent with the explanatory sequential design. All procedures complied with established ethical standards for research involving human participants (Israel and Hay, 2006).

### 3.6 Data analysis

Quantitative data were analyzed using SPSS 25.0. Descriptive statistics were computed to examine the distribution of psychological capital and classroom management self-efficacy scores. Internal consistency reliability was assessed using Cronbach’s alpha coefficients. Pearson product–moment correlation analyses were conducted to examine associations between psychological capital (total and subdimensions) and classroom management self-efficacy. Subsequently, multiple regression analysis was performed to test the predictive role of psychological capital components, with particular attention to the contribution of resilience, as hypothesized in the conceptual framework. Prior to regression analysis, assumptions of normality, linearity, multicollinearity, and homoscedasticity were examined. Variance inflation factor (VIF) values were below the recommended threshold, indicating no multicollinearity concerns. All statistical analyses followed standard assumptions and procedures recommended in quantitative research (Field, 2024).

Qualitative data were analyzed using thematic analysis, following the six-phase approach outlined by Braun and Clarke (2006), a widely used and flexible method for identifying patterns within qualitative data. Analysis combined deductive coding, informed by the conceptual framework (e.g., resilience, emotional regulation, stress tolerance), and inductive coding, allowing themes to emerge from participants’ reflections. To enhance trustworthiness, credibility, and rigor, strategies such as iterative coding, theme refinement, and alignment with quantitative findings were employed (Lincoln and Guba, 1985). Integration occurred at the interpretation level through the comparison of quantitative results and qualitative themes. Quantitative findings identifying resilience as a key predictor were elaborated by qualitative themes describing emotional regulation, persistence, and recovery from classroom setbacks. This integrative process enabled the development of meta-inferences, a key feature of mixed-methods research (Creswell and Clark, 2017).

### 3.7 Ethical considerations

The study was conducted in full compliance with internationally recognized ethical standards for research involving human participants and in accordance with the principles of the Declaration of Helsinki. Ethical approval was obtained from the Scientific Research Ethics Committee of Biruni University. The study was reviewed and approved in the committee meeting held on 12 December 2024, with the approval number 2024-BIAEK/05-234.

Participation was voluntary, and written informed consent was obtained from all participants prior to data collection. Anonymity and confidentiality were ensured throughout the research process. Participants were also informed of their right to withdraw from the study at any stage without penalty.

## 4 Results

### 4.1 Reliability and descriptive statistics

The internal consistency of the measurement instruments used in the study was examined using Cronbach’s alpha coefficients. The Psychological Capital Scale demonstrated high internal consistency (*α* = 0.91), indicating that the scale was reliable for the present sample. Reliability coefficients for the subdimensions were also satisfactory: self-efficacy (*α* = 0.88), hope (*α* = 0.86), resilience (*α* = 0.84), and optimism (*α* = 0.82). These values indicate that all subdimensions of psychological capital demonstrated satisfactory internal consistency for the present sample.

Similarly, the Teachers’ Classroom Management Self-Efficacy Scale (TCMSES) demonstrated high internal reliability (*α* = 0.89), supporting its suitability for assessing pre-service teachers’ perceived classroom management competence.

Descriptive statistics revealed that participants reported high levels of overall psychological capital (*M* = 4.12). Examination of the subdimensions indicated that the highest mean score was observed for hope (*M* = 4.28), whereas the lowest mean score was observed for resilience (*M* = 3.98). The mean score for classroom management self-efficacy was also high (*M* = 4.09), suggesting that pre-service English teachers generally perceived themselves as competent in managing classroom processes during practicum.

Table 1 shows that participants reported generally high levels of psychological capital. The highest mean score was obtained for *“I can cope with difficult tasks”* (*M* = 4.27), indicating strong perceived self-efficacy. Similarly, high scores for self-motivation and trying alternative strategies reflect strong hope- and agency-related beliefs. In contrast, lower mean scores were observed for resilience-related items, particularly *recovering quickly in difficult times* (*M* = 3.45) and *feeling completely confident about the future* (*M* = 3.40), compared to other psychological capital indicators. These findings indicate that participants perceived recovery from difficulties and adaptation to unexpected challenges as relatively more demanding.

**Table 1:** Descriptive statistics of psychological capital scale items (*n* = 55).

Item no	Item summary	M	SD
1	I can cope with difficult tasks	4.27	0.58
2	I try different ways to achieve success	4.25	0.62
3	I can motivate myself	4.25	0.64
4	I believe that I can achieve my goals	4.18	0.67
5	I do not give up in the face of obstacles	4.12	0.71
6	I look at the future with hope	4.05	0.74
7	I can generate solutions in difficult situations	3.98	0.81
8	I adapt to unexpected problems	3.72	0.86
9	I recover quickly in difficult times	3.45	0.90
10	I feel completely confident about the future	3.40	0.93

Table 2 indicates that participants perceived themselves as competent in fundamental classroom management practices such as establishing rules, maintaining order, and communicating effectively with students. However, comparatively lower mean scores were observed for managing student inattention (*M* = 3.74) and behavioral problems (*M* = 3.58), suggesting that disruptive behaviors represent the most challenging aspects of classroom management during practicum.

**Table 2:** Descriptive statistics of teachers’ classroom management self-efficacy scale (*n* = 55).

Item no	Item summary	M	SD
1	I can clearly establish classroom rules	4.07	0.65
2	I can maintain classroom order	4.05	0.66
3	I communicate effectively with students	4.03	0.69
4	I use class time effectively	3.97	0.72
5	I motivate students to participate	3.88	0.75
6	I can manage students’ inattention	3.74	0.81
7	I can manage behavioral problems	3.58	0.89

### 4.2 Quantitative findings: correlation analysis

To examine the relationship between psychological capital and classroom management self-efficacy, Pearson product–moment correlation analyses were conducted.

As shown in Table 3, the results indicate a strong, positive, and statistically significant relationship between pre-service English teachers’ psychological capital and their classroom management self-efficacy (*r* = 0.71, *p* < 0.001). This finding indicates that higher levels of psychological capital are associated with higher levels of perceived classroom management self-efficacy. The magnitude of the correlation suggests that psychological capital is strongly associated with pre-service teachers’ perceived classroom management competence during practicum.

**Table 3:** Relationship between psychological capital and classroom management self-efficacy (*n* = 55).

Variables	*r*	*p*
Psychological capital – classroom management self-efficacy	0.71	<0.001

As shown in Table 4, all subdimensions of psychological capital were positively and significantly associated with classroom management self-efficacy. Among these components, resilience exhibited the strongest relationship with classroom management self-efficacy (*r* = 0.74, *p* < 0.001), followed by self-efficacy (*r* = 0.66) and hope (*r* = 0.61). Optimism demonstrated a moderate yet statistically significant association (*r* = 0.52).

**Table 4:** Correlations between psychological capital subdimensions and classroom management self-efficacy (*n* = 55).

Subdimension	*r*	*p*
Self-efficacy	0.66	<0.001
Hope	0.61	<0.001
Resilience	0.74	<0.001
Optimism	0.52	<0.001

Importantly, these results indicate a clear pattern: although resilience had the lowest mean score among psychological capital components (*M* = 3.98), it showed the strongest association with classroom management self-efficacy. This finding suggests that resilience may be a contextually salient psychological resource in classroom management situations characterized by stress, uncertainty, and emotional demand. Moreover, these findings suggest that while all psychological capital components are positively related to classroom management self-efficacy, their relative strength varies, with resilience appearing as a more salient resource in practicum contexts.

### 4.3 Qualitative findings

Qualitative data obtained through open-ended reflective written responses were analyzed using thematic analysis. The analysis yielded three main themes reflecting pre-service English teachers’ experiences regarding the role of psychological capital in classroom management during the teaching practicum. These themes provide explanatory insight into the quantitative findings by illustrating how psychological resources, particularly resilience, are experienced and enacted in authentic classroom contexts.

#### 4.3.1 Theme 1: Resilience as a contextually salient resource in classroom management

Participants consistently described classroom management during practicum as emotionally demanding and stressful. They emphasized that their ability to remain calm, persistent, and emotionally regulated in the face of classroom challenges played a critical role in sustaining instructional continuity and classroom control.

“Sometimes the classroom becomes very noisy and I feel stressed, but I try to control myself andcontinuethelesson.”(P12).

“Even when students do not listen, I remind myself to stay calm and not to give up.” (P27).

“There were days when I felt unsuccessful, but I did not quit. I tried again in the next lesson.” (P41).

These accounts suggest that resilience may function as a protective psychological resource, helping pre-service teachers cope with stress, recover from setbacks, and maintain instructional continuity under challenging practicum conditions.

#### 4.3.2 Theme 2: Perceived competence but high implementation anxiety

Although participants generally reported confidence in their theoretical knowledge and pedagogical preparation, many expressed feelings of anxiety and uncertainty when applying this knowledge in real classroom settings. This theme reflects a discrepancy between perceived preparedness and enacted classroom practice during practicum.

“I know classroom management techniques, but I feel nervous when I have to use them.” (P8).

“Everything I learned seems easy in theory, but in practice I feel anxious.” (P19).

“I feel confident before entering the classroom, but once the lesson starts, I get nervous.” (P34).

These statements reveal that classroom management during practicum involves not only cognitive and instructional demands but also significant emotional challenges. The findings suggest that implementation anxiety may undermine perceived competence, highlighting the importance of psychological resources that support emotional regulation in authentic teaching contexts.

#### 4.3.3 Theme 3: The limits of hope and optimism without stress tolerance

Participants acknowledged the value of optimism and hope in motivating them to enter the classroom and sustain positive expectations about teaching. However, they emphasized that these resources were insufficient on their own when confronted with disruptive behaviors and emotionally demanding classroom situations.

“Being positive helps me start the lesson, but it does not solve classroom problems.” (P15).

“I am hopeful about becoming a good teacher, but sometimes the classroom environment is overwhelming.”(P22).

“Positive thinking is important, but managing stress is more important in the classroom.” (P49).

These responses indicate that effective classroom management during practicum appears to depend more strongly on resilience-based psychological resources, such as stress tolerance and emotional regulation, than on positive expectations alone. This theme provides qualitative support for the quantitative finding that resilience is more strongly associated with classroom management self-efficacy than optimism or hope.

Overall, the qualitative findings illustrate that while pre-service teachers possess positive beliefs and pedagogical knowledge, their classroom management experiences during practicum are shaped not only by pedagogical knowledge and positive beliefs, but also by their ability to cope with stress, regulate emotions, and persist despite challenges. The themes collectively underscore the contextually salient role of resilience in translating psychological capital into effective classroom management beliefs in real teaching contexts. Taken together, the qualitative themes help explain the quantitative pattern observed in the correlation analysis. While hope, optimism, and self-efficacy were positively associated with classroom management self-efficacy, participants’ reflections indicated that resilience became particularly visible in moments of classroom disruption, emotional pressure, and perceived loss of control. Thus, the qualitative findings elaborate the statistical results by showing how psychological capital is experienced as a lived resource during practicum. This integrated interpretation supports the view that psychological capital operates as a dynamic and context-dependent system of resources, in which different components may become more or less salient depending on situational demands encountered during teaching practicum.

## 5 Discussion

The present study examined the relationship between psychological capital and classroom management self-efficacy among pre-service English teachers during teaching practicum through an explanatory sequential mixed-methods design. The findings indicated that psychological capital was strongly associated with classroom management self-efficacy, supporting previous research that highlights the role of psychological capital in enhancing teachers’ professional functioning, wellbeing, and efficacy beliefs in demanding educational contexts (Luthans et al., 2006; Zhou and Zheng, 2022). The integration of quantitative and qualitative findings further provided explanatory insight into how psychological resources are experienced and mobilized in emotionally demanding practicum contexts, particularly in relation to stress, uncertainty, and classroom challenges.

These findings are also consistent with social cognitive theory (Bandura, 1997), which emphasizes that self-efficacy beliefs are shaped not only by instructional competence but also by individuals’ capacity to regulate emotions and cope with challenging situations. From this perspective, psychological capital can be understood as a set of adaptive psychological resources that support the development and enactment of classroom management self-efficacy in real teaching environments.

The discussion is structured in relation to the three research questions (RQ1–RQ3) and interprets the findings in light of psychological capital, self-efficacy, resilience, and stress-coping perspectives, with particular attention to the contextually salient role of resilience.

### 5.1 Psychological capital and classroom management self-efficacy (RQ1)

Addressing RQ1, the quantitative findings revealed a strong, positive, and statistically significant relationship between overall psychological capital and classroom management self-efficacy (*r* = 0.71, *p* < 0.001). This finding is consistent with Bandura’s (1997) self-efficacy theory, which emphasizes that individuals’ beliefs about their capabilities influence how they approach, persist in, and regulate their responses to challenging situations. It is further supported by prior research demonstrating that psychological capital is positively associated with self-efficacy, work engagement, and adaptive functioning in educational and organizational contexts (Luthans et al., 2006; Avey et al., 2009).

From a theoretical perspective, this result supports the conceptualization of classroom management self-efficacy as a psychologically grounded belief, shaped not only by pedagogical knowledge but also by individuals’ psychological resources (Slater and Main, 2020). Similar findings have been reported in teacher education research, indicating that teachers’ efficacy beliefs are closely linked to their emotional experiences and coping capacities in classroom settings (Ji et al., 2022). Therefore, the present findings extend existing literature by demonstrating that psychological capital appears to function as an important psychological foundation for effective classroom management during the emotionally demanding practicum period.

### 5.2 Differential roles of psychological capital components (RQ2)

In response to RQ2, the subdimension-level analyses revealed that all components of psychological capital—self-efficacy, hope, optimism, and resilience—were significantly associated with classroom management self-efficacy. However, resilience showed the strongest association with classroom management self-efficacy (*r* = 0.74, *p* < 0.001), despite having the lowest mean score among the components. The present study identifies this pattern as a “resilience paradox”: although resilience received the lowest mean score among psychological capital components, it showed the strongest association with classroom management self-efficacy. This finding suggests that resilience may become more functionally salient under practicum-related stress, where pre-service teachers are required to regulate emotions, recover from classroom setbacks, and sustain instructional control despite uncertainty. This finding aligns with resilience theory, which conceptualizes resilience as a dynamic adaptive process that becomes particularly salient under conditions of stress and adversity (Masten and Reed, 2002). This interpretation is also compatible with stress and coping theory, which emphasizes that individuals’ responses to demanding situations depend on how they appraise and manage stressors (Lazarus and Folkman, 1984). In practicum classrooms—where pre-service teachers face uncertainty, disruptive behaviors, and emotional strain—resilience may become particularly salient in enabling adaptation under stress, while operating alongside other Psychological capital components to translate into effective classroom management beliefs. Recent meta-analytic evidence also supports the strong association between teacher resilience and self-efficacy, particularly in early-career and high-stress teaching contexts (Mu et al., 2024).

While hope and optimism were reported at high levels, their associations with classroom management self-efficacy were comparatively weaker. This finding suggests that positive expectations and goal-oriented motivation may facilitate initial engagement with teaching tasks but are insufficient for sustaining classroom control in emotionally demanding situations. Similar conclusions have been drawn in recent studies, which emphasize that positive affective states require emotion regulation and stress tolerance to support sustained professional functioning (Zhou and Zheng, 2022; Zhang and Zhang, 2025).

### 5.3 Explanatory role of psychological capital in practicum experiences (RQ3)

Addressing RQ3, the qualitative findings provided explanatory insight into how pre-service teachers experience and enact psychological capital—particularly resilience—when managing classroom challenges during practicum. Participants consistently described classroom management as emotionally demanding and emphasized the importance of remaining calm, persistent, and emotionally regulated. These narratives suggest that resilience appears to function as a protective psychological resource that supports adaptive functioning under practicum-related stress, enabling pre-service teachers to recover from setbacks and maintain instructional continuity.

The qualitative findings revealed a gap between theoretical competence and practical self-confidence, as many participants reported experiencing anxiety when applying classroom management strategies in real classroom settings. This finding complements the quantitative results by demonstrating that classroom management self-efficacy is shaped not only by perceived instructional competence but also by emotional readiness. Consistent with prior research, psychological capital appears to function as a critical psychological resource that supports teachers’ capacity to cope with stress and adapt to challenging professional demands, thereby facilitating the translation of theoretical knowledge into effective classroom practice (Luthans et al., 2006; Avey et al., 2009; Ortega-Maldonado and Salanova, 2017). In this sense, psychological capital may be understood as a mechanism that enables individuals to mobilize their cognitive and emotional resources in ways that bridge the gap between knowing and doing in complex teaching environments.

The qualitative findings further suggest that classroom management self-efficacy is closely connected to emotion regulation. Participants’ reflections showed that maintaining calmness, managing anxiety, and recovering from unsuccessful teaching moments were central to their classroom management experiences. This aligns with emotion regulation perspectives, which emphasize that individuals’ ability to regulate emotional responses shapes their functioning in demanding social and professional contexts (Gross, 1998).

Furthermore, participants’ emphasis on stress tolerance over optimism and hope provides qualitative support for the resilience paradox identified in the quantitative phase. While optimism and hope motivated participants to enter the classroom, resilience was perceived as particularly relevant under practicum-related stress and real classroom disruptions. This finding reinforces contemporary views that resilience is contextually salient to sustaining teacher effectiveness in challenging teaching environments (Mieres-Chacaltana et al., 2025).

Importantly, the convergence between quantitative and qualitative findings strengthens the explanatory power of the study, as qualitative insights help clarify why resilience shows a stronger association with classroom management self-efficacy in practicum contexts.

Overall, the integrated findings support a context-sensitive interpretation of psychological capital. Rather than positioning resilience as a universally dominant component, the results suggest that resilience may become particularly salient in practicum contexts marked by emotional pressure, classroom uncertainty, and implementation anxiety. This interpretation offers a more balanced understanding of psychological capital as a dynamic system of resources contributing to classroom management self-efficacy. This interpretation also contributes to a more nuanced understanding of how psychological resources operate in real teaching contexts.

## 6 Conclusion

This study investigated the relationship between psychological capital and classroom management self-efficacy among pre-service English teachers during teaching practicum through an explanatory sequential mixed-methods design. The findings demonstrated that psychological capital is strongly associated with classroom management self-efficacy and that this relationship becomes particularly meaningful in emotionally demanding classroom contexts.

A key contribution of the study is the identification of a resilience paradox, whereby resilience, despite being comparatively less developed, shows the strongest association with classroom management self-efficacy. This finding suggests that resilience may become particularly salient under practicum-related stress, supporting pre-service teachers’ ability to regulate emotions, recover from challenges, and sustain instructional effectiveness.

Overall, the results support a context-sensitive understanding of psychological capital as a dynamic system of interrelated resources. Rather than functioning as isolated traits, these psychological resources appear to operate in interaction, with their relative importance shaped by situational demands. By integrating quantitative and qualitative findings, the study provides a more comprehensive explanation of how psychological readiness contributes to classroom management experiences during practicum.

## 7 Implications and recommendations

The findings suggest that effective classroom management during practicum depends not only on pedagogical knowledge but also on pre-service teachers’ psychological readiness. Psychological capital and self-efficacy appear to function as complementary, context-sensitive resources that support adaptation in emotionally demanding classroom environments.

Accordingly, teacher education programs may benefit from integrating both psychological capacity-building and self-efficacy development into practicum preparation. In particular, practices such as reflective supervision, peer support, and stress-regulation activities may enhance pre-service teachers’ ability to cope with classroom challenges while strengthening their confidence in managing instructional processes.

In addition, mentoring processes should address emotional experiences alongside instructional performance. Providing emotionally responsive feedback may help reduce implementation anxiety and support the development of classroom management self-efficacy. Incorporating formative assessments of psychological capital and self-efficacy may further enable early identification of support needs.

## 8 Limitations and future research

This study has several limitations. The sample was limited to 55 pre-service teachers from a single program, which may restrict generalizability. The reliance on self-report measures may also introduce subjectivity. Future research should include larger and more diverse samples and use multiple data sources, such as observations and mentor evaluations. Longitudinal designs are also needed to examine how psychological capital develops over time. Finally, intervention-based studies may explore how resilience and related psychological resources can be enhanced to improve classroom management and teacher wellbeing during practicum.

## Data Availability

The raw data supporting the conclusions of this article will be made available by the authors, without undue reservation.
